# Uncovering True Cellular Phenotypes: Using Induced Pluripotent Stem Cell-Derived Neurons to Study Early Insults in Neurodevelopmental Disorders

**DOI:** 10.3389/fneur.2018.00237

**Published:** 2018-04-16

**Authors:** James J. Fink, Eric S. Levine

**Affiliations:** Department of Neuroscience, University of Connecticut School of Medicine, Farmington, CT, United States

**Keywords:** neurodevelopmental disorders, autism spectrum disorders, electrophysiology, synaptic transmission, induced pluripotent stem cells

## Abstract

Animal models of neurodevelopmental disorders have provided invaluable insights into the molecular-, cellular-, and circuit-level defects associated with a plethora of genetic disruptions. In many cases, these deficits have been linked to changes in disease-relevant behaviors, but very few of these findings have been translated to treatments for human disease. This may be due to significant species differences and the difficulty in modeling disorders that involve deletion or duplication of multiple genes. The identification of primary underlying pathophysiology in these models is confounded by the accumulation of secondary disease phenotypes in the mature nervous system, as well as potential compensatory mechanisms. The discovery of induced pluripotent stem cell technology now provides a tool to accurately model complex genetic neurogenetic disorders. Using this technique, patient-specific cell lines can be generated and differentiated into specific subtypes of neurons that can be used to identify primary cellular and molecular phenotypes. It is clear that impairments in synaptic structure and function are a common pathophysiology across neurodevelopmental disorders, and electrophysiological analysis at the earliest stages of neuronal development is critical for identifying changes in activity and excitability that can contribute to synaptic dysfunction and identify targets for disease-modifying therapies.

## Introduction

In addition to their prevalence and impact on the individual patient, neurological disorders place a large burden on the world population both in economic impact (1) and emotional toll on families. For these reasons, there is an urgent need to develop effective treatments for managing these devastating disorders. To date, the use of animal models to study how disease-causing genetic mutations lead to cellular, molecular, and circuit-level pathophysiology in the brain has led to many important discoveries and a better understanding of disease mechanisms. However, despite these advancements, the development of effective treatments for a wide-variety of neurological disorders remains elusive.

These shortcomings have often been attributed to species differences, including genetic complexity and/or incomplete modeling of relevant behavioral phenotypes, resulting in potential treatments that are promising at early stages in animal models but fail during clinical trials on human subjects. This situation has led to a push for the use of human cells for studying neurological disease pathology and validating therapeutic targets (2, 3). Study of human neurons has generally relied on postmortem tissue, which contains unique biological information but is limited by sample size, especially for rare neurogenetic disorders, and variable sample quality. Alternatively, non-invasive imaging techniques can provide some insight into underlying pathology but lack cellular-level spatial resolution, and the use of aborted fetal brain tissue and human embryonic stem cells are associated with ethical controversies (4–6).

The 2007 discovery that somatic cells from human subjects can be reprogrammed into induced pluripotent stem cells (iPSCs) *via* the overexpression of a distinct set of transcription factors has provided an entirely new avenue for studying disease pathology in neurogenetic disorders (7). Using this technique, patient-specific cell lines can be differentiated into neurons that can serve as a valuable complement to animal models for identifying primary phenotypes at the cellular and molecular level. These samples can be collected from large patient groups, banked, and widely distributed for studies with greater statistical power.

It is clear from animal models and postmortem studies that impairments in synaptic structure and function are a common pathophysiology across disorders (8–10). These findings highlight the importance of synaptic activity in normal brain function and have identified key molecular targets underlying certain forms of pathology, but in many cases these studies have failed to explain how synaptic dysfunction can lead to different neurological disorders. It is likely that synaptic deficits are often times a secondary phenotype that is a consequence of primary changes to neuronal function and excitability early in development. These primary phenotypes could trigger homeostatic responses in neurons and glia that could then result in further changes to excitability including action potential (AP) firing, synaptic communication, and long-term activity-dependent synaptic plasticity. In fact, a recent review has already proposed a similar theme of pathophysiology in relation to autism spectrum disorder (ASD), which is commonly referred to as a “synaptopathy” (11).

In this review, we explore how iPSC-derived neuron technology will help in identifying primary cellular phenotypes associated with neurogenetic disorders, specifically neurodevelopmental disorders, and how these studies might better inform future investigation into pathophysiology and disease treatments and therapies. In particular, we will focus on the use of electrophysiological analysis of iPSC-derived neurons, as understanding the dynamic function of neurons during disease development will ultimately lead to insight into the behaviors and symptoms associated with these neurological disorders.

## Mouse Models of Neurodevelopmental Disorders

Autism spectrum disorder consists of a collection of heterogenous neurodevelopmental disorders characterized by impairments in social interactions, including language development, as well as restricted interests and repetitive behaviors (12, 13). The prevalence of ASD has been estimated at 1 in 68 (14), and despite the extensive research on understanding the underlying causes of this disorder, the results have been underwhelming (15). Part of this issue is the heterogeneity, not only of the breadth and severity of symptoms across patients, but of the complex genetic alterations associated with ASD (16–18).

To overcome this, recent efforts have focused on studying syndromic forms of autism including single-gene disorders, such as Rett syndrome, Fragile X syndrome (FXS), tuberous sclerosis complex (TSC), and Angelman syndrome (AS), as well as copy number variations in regions 15q11-q13 (Dup15q), 16p11.2, 22q11.2, and others (19). Knowing the causative genes and genomic regions associated with this group of syndromic ASDs allows a more powerful study of the underlying pathophysiology as compared with idiopathic ASD. These studies strongly suggest that heterogenous synaptic deficits underlie these disorders (15, 20). Importantly, animal models based on genes that have been strongly associated with ASD, such as SHANK3 and Neurexin-1 (NRXN1), further support the idea that autism is a disorder of defective synapses (21–23). General findings from these various mouse models are discussed below.

### 15q11-q13-Associated Disorders: as and Dup15q

Opposite changes in the chromosome 15q11-q13 region give rise to two disorders associated with autistic phenotypes. Maternal deletion of this region gives rise to AS, a neurodevelopmental disorder characterized by inappropriate laughter, ataxia, and seizures. Some patients also present with autistic features such as developmental delay, intellectual disability, and a lack of language (24). Maternal duplication of this region results in chromosome 15q duplication syndrome (Dup15q), a disorder in which autism is highly penetrant (25). These disorders result from chromosomal changes specific to the maternal allele because the 15q11-q13 region is subject to regulation by genomic imprinting, an epigenetic process resulting in allele-specific gene expression. In the 15q11-q13 region, several genes are exclusively expressed from one parent allele or the other.

One of these genes, *UBE3A*, is imprinted in a cell type-specific manner. In particular, in neurons, *UBE3A* is solely expressed from the maternal allele, due to paternal silencing (26–28). AS is specifically caused by loss of *UBE3A*, as point mutations in *UBE3A* are sufficient to cause the disorder, accounting for ~15% of AS cases (29). UBE3A is an E3 ubiquitin ligase responsible for tagging target proteins for degradation by the proteasome, and some of these targets may be important synaptic proteins. Findings in animal models of these disorders support the dysfunctional synapse hypothesis described earlier (29). For instance, mice lacking maternal *UBE3A* have impaired activity-dependent synaptic maturation, decreased inhibitory activity onto pyramidal neurons, and impaired synaptic plasticity (30–32). Mouse models of Dup15q suggest similar themes of disruption. For instance, in the Dup15q mouse model with large duplications of the syntenic region, impairments in spine turnover and cerebellar synaptic plasticity have been observed (33, 34). However, these cellular phenotypes, along with relevant behavioral phenotypes, are only observed in paternal duplication mice, whereas only maternal duplication results in Dup15q in patients (35). In another mouse model, increased levels of *UBE3A* are linked to decreased glutamatergic signaling, suggesting aberrant synaptic activity may be involved in the pathophysiology of Dup15q (36). The maternal inheritance of Dup15q and the phenotypic overlap with AS strongly implicate *UBE3A* as a causative gene in Dup15q. However, GABAR subunits and other genes linked to neuronal function are duplicated in this region and are probably also involved. The difficulty in developing animal models that accurately mimic large duplications and/or changes to multiple genes provides a strong argument for the use of human cells with the actual disease-causing mutations for modeling Dup15q and other polygenic syndromes.

### Fragile X Syndrome

Fragile X syndrome, which is also associated with an autism phenotype, is due to CGG repeat expansion within the *FMR1* gene. This results in hypermethylation and therefore *FMR1* silencing (37). Animal models and postmortem human nervous tissue have consistently shown abnormal dendritic spine structure and increases in spine number (38). In addition, there may be deficits in GABAergic signaling leading to imbalances in excitatory and inhibitory activity (39). The *FMR1* gene encodes a protein (FMRP) that acts as a translational repressor, regulating the translation of many important synaptic proteins at or near dendritic spines (40). For instance, some FMRP target mRNA’s include PSD95, Arc, CaMKII, and AMPAR subunit mRNAs (41). Arc and CaMKII have also been tied to UBE3A and AS pathophysiology. These findings suggest aberrant synaptic signaling most likely underlies at least some aspects of FXS.

### Rett Syndrome

Rett syndrome (RTT)—a neurodevelopmental disorder in which affected females develop normally until 1–2 years of age but subsequently show developmental decline, motor abnormalities, and autistic symptoms—is another disorder in which it has been clearly shown that synaptic signaling is abnormal (42). RTT arises from mutations in the X-linked *MECP2* gene, responsible for producing a methyl-CPG-binding protein. This protein controls the expression of many other proteins by binding to their promoters (43). Some examples include BDNF, UBE3A, and GABAB3 (44). BDNF is important for activity-dependent regulation of dendritic development and synaptic activity and plasticity and BDNF signaling is impaired in AS mice (45, 46). UBE3A and GABAB3 are also associated with 15q11-q13 disorders and are core components of synaptic function. MECP2 itself is abundant in neurons, and changes in this protein have been linked to reduced dendritic spine numbers, impaired long-term potentiation (LTP), and decreased excitatory and inhibitory synaptic transmission, deficits that are almost analogous to those identified in FXS models (47, 48).

### Other ASD-Associated Mutations

Similar abnormalities have been found in models of TSC, Timothy syndrome (TS), Phelan–McDermid syndrome (PMS) (SHANK3 mutations), and neuroligin and neurexin mutant mice (15). The latter two (neuroligins and neurexins) are genes whose mutations have been identified in patients with ASD (23). Changes in *TSC* genes lead to changes in dendritic spine length and density as well as synaptic plasticity (49). SHANK3 mutations have been linked to synapse formation and dendritic spine regulation, and mouse models of SHANK3 mutations have ASD-relevant behaviors, including social impairments (50). Neuroligins, which interact with SHANK, and neurexins, have also been linked to ASD-relevant behaviors in mice. Not surprisingly, mutations in these genes lead to disruptions in synaptic transmission.

It is clear from the above discussion of animal models of ASD-related disorders that abnormal synaptic activity and plasticity are linked to impaired brain function in association with the underlying pathophysiology of these disorders. Due to the difficulty of associating mouse neural development with the appropriate corresponding stages of human neural development, it is less clear when these types of synaptic dysfunction arise in human brain tissue. Again, the possibility also exists that such synaptic changes may be downstream consequences of intrinsic functional changes in neurons or glia at embryonic stages of development, or could represent compensatory homeostatic shifts in response to such primary deficits. For this reason, studying the *in vitro* development of human neurons harboring disease-causing genetic insults provide us with a richer understanding of the pathophysiology of ASD.

## The Promise of iPSCs for Modeling Neurodevelopmental Disorders

Although animal models of disease are well suited for the study of monogenetic disorders, many genetic forms of ASD are due to larger deletions or duplications encompassing multiple genes that are not easily modeled in animals. The discovery of iPSC technology and the ability to generate specific subtypes of neurons from patient-derived samples allows for the accurate modeling of these disorders at a genetic level. Furthermore, iPSC models provide a means for identifying physiological deficits *in vitro* in human neurons roughly equivalent to gestational stages of development, as previously discussed (4) that can lead to synaptic dysfunction in mature neurons. Below we summarize some of the initial work using iPSC-derived neuronal models and the promises they hold for building on in the future.

### 15q11-q13 Disorders

As described earlier, AS results from maternal loss of the *UBE3A* gene, which is paternally silenced in neurons by a long non-coding antisense transcript that is not made on the maternal allele due to methylation. Thus, to accurately model this disorder with iPSC-derived neurons, these neurons would also have to show the appropriate imprinting of this genetic region. In one of the earlier studies of iPSC models of neurodevelopmental disorders, Chamberlain and colleagues derived neurons from subjects with AS and Prader–Willi syndrome (associated with paternal chromosome 15q deletion) (51). They found that correct imprinting occurs during the differentiation of iPSCs into neurons, thus silencing paternal *UBE3A*, and neurons from AS subjects are missing UBE3A protein. Although it was shown that these neurons were functional, as they fired APs and had synaptic activity, no comparisons were made regarding the electrophysiological phenotypes between patient cell lines and controls. A more recent study, discussed below, has in fact identified functional deficits in neuronal and synaptic maturation in AS neurons (52) and others have further explored AS and PWS-associated imprinting in both iPSC-derived neurons and non-neuronal cells (53–55).

Induced pluripotent stem cell models have also been used to examine changes in gene expression in neurons from patients with either AS (15q11-q13 deletion) or Dup15q, which despite opposite genetic causes, present with overlapping clinical phenotypes. Interestingly, copy number did not consistently predict gene expression levels, and most of the genes that were differentially expressed in both AS and Dup15q neurons compared with controls changed in the same direction (56). A recent follow-up study, also discussed below, has uncovered electrophysiological deficits in Dup15q neurons that are distinct from the AS neuronal phenotype (57).

### Rett Syndrome

Several studies have developed iPSC-derived neuronal models of Rett syndrome. One of the earliest and most impressive studies capitalized on non-random X-linked inactivation to derive isogenic pairs of neurons from Rett syndrome patients and observed a synaptic deficit as measured by decreases in the number of vGLUT puncta and a decrease in spine density in lines containing the mutation. This morphological deficit was confirmed by electrophysiological recordings, which revealed decreases in both the frequency and amplitude of spontaneous excitatory synaptic currents (58). The synaptic deficit could be rescued by treatment with IGF1, which has been shown to improve RTT-associated behaviors in a mouse model (59). Another study by the same group observed an impairment in the developmental switch from excitatory to inhibitory GABA signaling due to reduced expression of the KCC2 chloride transporter (60). Additional studies of RTT pathophysiology have also used isogenic cell lines. These studies observed a smaller soma size and disruptions in developmental maturation, although this latter conclusion was based on decreases in various neuronal markers as measured by qPCR and immunocytochemistry (61–63).

Similar themes of developmental disruption *in vitro* have been confirmed in iPSC-derived neuron models with additional RTT- and seizure-associated gene mutations including CDLK5 (64–66) and FOXG1 (67). In mouse hippocampal neurons, CDLK5 is localized to excitatory synaptic compartments and is important for dendritic spine function. This was confirmed in human cells as iPSC-derived neurons with CDLK5 mutations had reductions in dendritic spine number (66). The role of GluD1, a glutamate receptor subunit that acts as a synaptic adhesion molecule, has also been examined in RTT-associated MECP2, CDLK5, and FOXG1 mutations (64, 67). Changes in GluD1 expression in iPSC-derived neurons were associated with changes in excitatory and inhibitory synapse number as measured by expression levels of synaptic markers; however, synaptic function/dysfunction was not examined electrophysiologically. With regard to electrophysiological characterization, iPSC-derived neurons with MECP2 isoform-specific mutations have significantly increased input resistance and decreased capacitance, consistent with the decreased soma size described above (68). AP firing, voltage-gated sodium and potassium currents, and spontaneous synaptic activity were all altered in MECP2 mutant neurons. Electrophysiological measurements of iPSC-derived neurons from a mouse model of RTT (69) found similar results, with decreases in AP firing and synaptic activity.

Interestingly, both *MECP2* duplications as well as deletions result in syndromes with overlapping phenotypes in patients, analogous to the 15q11-q13 region. iPSC-derived neurons from patients with *MECP2* duplications also develop synaptic impairments, but with elevated levels of synaptic markers and increased dendritic complexity as measured by Sholl analysis (70). Network activity monitored by multielectrode arrays (MEAs) also revealed increases in burst activity and neuronal synchrony.

Induced pluripotent stem cell models have also yielded insights into the role of glia in the pathophysiology of neurological disorders. For instance, both RTT mouse astrocytes and RTT iPSC-derived astrocytes have disrupted microtubule transport (71), which has implications for synaptic maintenance and additional astrocyte functions. A non-cell-autonomous role for iPSC-derived astrocytes in RTT has also been established, as either the presence of astrocyte-conditioned media or astrocyte coculture with iPSC-derived neurons negatively impacts neuronal morphology and function, independent of changes to neuronal intrinsic properties, which are also known to contribute to changes in morphology. This suggests distinct mechanisms for morphological changes in neurons (72). This study, however, only provided limited insight into alterations in synaptic function.

### Fragile X Syndrome

A large number of studies have used iPSC-derived neurons to model FXS (4). Most have provided proof-of-concept studies that validate appropriate FMRP1 expression and CGG repeat lengths and focus on the development of various high-throughput assays for rescuing FMR1 expression (73–78). These studies have also confirmed many of the broad phenotypes previously established in mouse models of FXS. For instance, it has been shown that iPSC-derived neurons have disrupted neuronal differentiation, including decreases in both the length and number of neuronal processes (76, 78). Gene expression analysis confirmed disruptions in neuronal differentiation as important axon guidance genes such as SLIT and ROBO were significantly decreased in FXS-derived neurons, which was mediated by the REST complex (79). iPSC-derived neurons have also been produced from patients with Fragile X-associated tremor/ataxia, a disorder that results from premutation expansions. Similar to typical FXS, these neurons also have reduced synapse number as measured by loss of PSD95 expression and reduced synaptic puncta, as well as decreased neurite length. These cells also had increased frequencies and amplitudes of calcium transients, suggesting elevated network activity (80).

Unlike with RTT iPSC studies, very little electrophysiology has been used in the study of iPSC-derived FXS neurons, despite the important electrophysiological phenotypes established in the mouse model. In contrast to this, there have been important studies using electrophysiological characterization of hESC-derived neuron models of FXS. These studies have confirmed similar reductions in neurite number and length as observed in iPSC studies, but have also measured impaired AP firing, showing that FXS neurons have immature APs characterized by small amplitudes and long durations (81). This was reflected by significantly reduced inward sodium currents. Frequency and amplitude of spontaneous synaptic events were also reduced in these cells. GABA receptor physiology has also been studied using electrophysiology by patching neurons and applying GABA locally (82). Two types of responses to GABA puff were observed: (1) a non-desensitizing, bicuculline-sensitive, mature response and (2) a desensitizing, bicuculline-insensitive immature response. Control neurons switched to the more mature response as they developed, whereas FXS neurons showed predominantly immature responses.

### Timothy Syndrome

Timothy syndrome results from mutations to the L-type calcium channel Cav1.2, which makes it a “channelopathy” associated with autism, thus electrophysiological characterization of TS neurons is highly important. Studies on iPSC neurons derived from TS patients observed impaired activity-dependent expression of genes related to ASD and calcium signaling (83, 84), as well as reduced expression of upper and lower cortical layer markers. APs and calcium transients also had longer durations, suggesting disrupted calcium channel inactivation. CaMKII, an important synaptic protein involved in LTP, was decreased in TS neurons and has also been linked to disrupted synaptic plasticity in AS mouse models. Primary cortical neurons from TS mice as well as TS iPSC-derived neurons also show disrupted dendritic motility in response to depolarization. Control neurons responded to KCl with dendritic extension, whereas TS neurons responded to KCl with dendritic retraction. As KCl is a manipulation commonly used to mimic “increased activity” in neuronal networks, this suggests TS neurons respond abnormally to changes in neuronal network activity (85).

### Other Autism-Associated Mutations

Disruptions of important synaptic molecules and scaffolding proteins, often associated with ASD and/or neurodevelopmental disorders, also lead to neuronal and synaptic pathophysiology in iPSC-derived neurons. Importantly, many of the papers in this category provide comprehensive electrophysiological characterization including investigation of both intrinsic properties and synaptic activity. First, PMS, caused by loss of the synaptic scaffolding protein SHANK3 has been modeled using both iPSC and hESC-derived neurons. These studies replicated findings of synaptic impairment and increased input resistance in neurons associated with SHANK3 mutations in mouse models. In contrast to the minimal electrophysiology carried out in other iPSC-derived neuron models of disease, these studies examined AP firing, spontaneous synaptic activity, evoked AMPAR and NMDAR responses, and responses to focal AMPAR and NMDAR stimulation, paired with extensive molecular and immunocytochemical characterization. As with RTT, IGF1 treatment could rescue the PMS phenotypes (86, 87).

hESC-derived neurons have also been used to study mutations in the synapse-associated proteins syntaxin-binding protein 1 (STXBP1), NRXN1, and L1CAM. STXBP1 is an important presynaptic protein associated with seizures. Mutations to STXBP1 in hESC-derived neurons resulted in significant decreases in spontaneous and evoked synaptic activity due to decreased neurotransmitter release, whereas input resistance, capacitance, and AP firing were unchanged (88). NRXN1 mutations also resulted in significant decreases in synaptic activity despite normal intrinsic properties and normal dendritic morphology and synapse number (89). L1CAM mutations, by contrast, caused significant decreases in axonal length and branching but also dendritic length, dendritic branching, and axon initial segment length. These changes in axon properties were reflected by significantly reduced inward Na^+^ currents and excitability in these cells (90). No changes were observed in number of synaptic puncta, input resistance, or cell capacitance.

Induced pluripotent stem cell-derived neuron models of additional ASD-associated mutations include TRPC6, CHD8, and FOXG1 (91–93). These mutations are associated with changes in gene expression linked to neurite growth, cell adhesion, and neural development (92, 93). TRPC6 mutations caused reduced synaptic puncta density and decreased dendritic length and branching. Interestingly, levels of TRPC6 can be changed by MECP2, whose mutation results in strikingly similar dendritic phenotypes, as discussed above. Although electrophysiological properties were not extensively characterized, TRPC6 mutant neurons fired APs and displayed voltage-gated inward and outward currents.

Finally, using a specific endophenotype of macrocephaly to select patients, a recent study uncovered a role for Wnt signaling in the pathophysiology of iPSC-derived neurons from idiopathic ASD patients. This study found changes in gene expression related to sensory perception, but also related to cell adhesion molecules (94). Similarly, patient-derived neurons displayed reduced dendritic branding and synaptic puncta density. Recordings using MEAs revealed decreased spiking and network bursting, which could be rescued by IGF1.

## Electrophysiological Analysis of iPSC-Derived Neurons

Several studies have provided extensive electrophysiological characterization of iPSC-derived neurons including addressing issues of variability across cell lines, multiple clonal lines, and differentiations, as well as benchmarks for maturity. These studies have been carried out both in the context of disease modeling but also in control iPSC-derived neurons. Such studies are important as understanding the functional consequence of genetic disruptions on neuronal communication will ultimately better inform our understanding of neural pathophysiology. These papers characterize AP firing, synaptic activity, and sometimes other intrinsic properties during a brief developmental window, usually during early *in vitro* development, and with limited numbers of lines.

A series of papers has focused on iPSC-derived neurons in relation to alcohol dependence, FKBP5 regulation, and glucocorticoid activation (95–97). These cells express voltage-gated inward and outward currents, fire APs in response to current injection, respond to puff application of glutamate and GABA, and display spontaneous synaptic activity. These studies found significant differences in ethanol-induced gene expression in iPSC-derived neurons from controls versus patients with alcohol dependence.

Induced pluripotent stem cell-derived neurons from members of a family with DISC1 mutations, which are associated with psychiatric disorders, found dysregulation of synaptic markers as measured by RNA sequencing as well as significantly reduced synaptic puncta as measured by immunocytochemistry (98). These findings were supported by electrophysiological recordings that showed decreased spontaneous synaptic activity at 4 and 6 weeks *in vitro*. This synaptic impairment was due to disrupted neurotransmitter release at the presynaptic terminal.

It has been established, using karyotyping and gene expression analysis, that iPSC-derived neurons from trisomy 21 patients, maintain trisomy (99). This genetic disruption was associated with significantly reduced frequencies of spontaneous synaptic activity as measured by electrophysiology. AP firing was not different between controls and patients. Other studies have shown increases in spiking and calcium transients in Williams syndrome-derived neurons complemented by gene expression and morphological studies, and that neurons derived from Alzheimer’s patients mature into functional neurons, as measured by the presence of AP firing and synaptic activity, despite the presence of AD cellular pathology (100, 101). It should be noted that this latter study did not perform in-depth electrophysiological analysis of genotypic differences. Nonetheless, this investigation provides insight into cellular dysfunction in these diseases that complements the genetic and molecular data provided in these studies.

Other studies have used electrophysiology to uncover cellular phenotypes. For example, patch clamp and MEA studies uncovered a hyperexcitable phenotype in iPSC-derived neurons from ALS patients with SOD1 mutations (102). Hyperexcitability was characterized by increases in both spontaneous and induced AP firing. A significant decrease in the delayed rectifier potassium current was also observed, which supports alterations in AP firing.

Two other studies have provided strong electrophysiological analysis of iPSC-derived neurons from patients with Dravet syndrome. Dravet syndrome is caused by mutations in the SCN1A gene, which codes for the Nav1.1 channel, and patients with these mutations present with intractable epilepsy in childhood. Mutations to Nav1.1 increased sodium current density in both pyramidal and bipolar neurons resulting in enhanced AP firing and burst activity (103). By contrast, a study that derived neurons from twins with Dravet syndrome found that sodium current density and AP firing were reduced in inhibitory neurons and unchanged in excitatory neurons (104).

## Electrophysiological Development and Maturation

To uncover developmental cellular phenotypes, it is critical to establish protocols for maturation *in vitro* that produced neurons that express mature AP firing, spontaneous synaptic transmission, and activity-dependent synaptic plasticity. Furthermore, developmental analysis of neuronal function *via* electrophysiology is required to differentiate early primary insults from homeostatic responses in late *in vitro* development. Several iPSC-derived neuron studies have examined electrophysiological maturation and benchmarks of maturity across time in culture, as reviewed below:

- Using a protocol designed to generate electrophysiologically mature neurons, 70–80% of recorded cells fired repetitive APs in response to current injection. Cells had ~1 GΩ input resistance and ~−50 to −60 mV RMP (105). AP features include >60 mV amplitude and a <3 ms half-width (HW), indicating mature APs. Greater than 50% of these cells fired spontaneous APs and 60–80% of neurons were synaptically active. Frequency of synaptic activity was between 0.5 and 1.5 Hz. Another recent study has performed similar electrophysiological analysis of iPSC-derived neurons (106).
- Another study has shown that input resistances go down with age, while cell capacitance goes up. Neuronal RMP becomes more hyperpolarized (−45 to −70 by 70 days in culture) and 100% of neurons firing >1 AP by 42 days in culture. AP amplitudes were between 80 and 100 mV and the average HW was <3 ms. 100% of neurons were synaptically active by >20 days *in vitro*. Frequency of spontaneous synaptic activity reached ~1 Hz at >50 days *in vitro*. This study also showed responses to AMPA, Kainate, and NMDA application (107).
- With regards to electrophysiological maturation in the context of disease, one study has performed a strong electrophysiological analysis of control neuron maturation as compared with ALS patient-derived neurons (108). Most neurons fired single APs with current injection across development with only 10–15% of cells showing repetitive firing. In this study, ~20% show no firing. Although these features were measured over time in culture, little maturation of these features was observed. However, across development, ALS neurons show greater extent of immaturity. About 40% of control neurons were synaptically active with little to no developmental maturation. Significantly less activity was observed in ALS neurons at later stages in culture. Glutamate, GABA, and glycine application responses were also measured.
- Electrophysiological development of iPSC-derived neurons plated on either laminin or astrocytes for up to 4 months showed maturation of electrophysiological properties over time (109). These included maturation of AP firing as measured by decreased AP HW and increased AP amplitudes as well as a reduction in input resistance, increase in capacitance, and increased intrinsic currents and synaptic event frequency. Increasing dendritic complexity was also observed across time in culture. This maturation was significantly better across all features when iPSC-derived neurons were cultured on astrocytes. For example, synaptic frequency was increased threefold to fourfold. This paper also shows that inhibitory synaptic activity matures more slowly than excitatory synaptic activity and that iPSC-derived neurons can respond to GABA, glutamate, and NMDA application. A more recent study has also used astrocyte cocultures to look at glutamatergic and GABAergic neurons to study excitatory/inhibitory balance, which has been shown to be an important role player in autism pathophysiology (110).
- Additional studies have developed a protocol that directly converts fibroblasts into neurons *via* the overexpression of neural-lineage transcription factors (111, 112). These induced neurons fire APs both spontaneously and in response to current injection. The amplitudes of these APs are similar to values described above and the RMP of these cells becomes more hyperpolarized with time in culture. Input resistance decreases from >1 GΩ early in development to less than 500 MΩ in late development, and capacitance increases over time. APs become more and more repetitive as *in vitro* development proceeds, and although synaptic development is not as closely studied in this paper, the traces depicted in this study indicate the presence of increased levels of spontaneous synaptic activity.
- Developmental analysis of hESC-derived neurons shows that very young cells during the neuroepithelial rosette stage show increases in Na^+^ current with time in culture, with little change in outward K^+^ currents (113). hESC-derived neurons later in development, however, show decreasing Na^+^ and K^+^ currents, increasing input resistances, and more depolarized RMP across time. They also classify AP firing into four categories ranging from immature passive responses to more mature repetitive firing, and that these neurons are very heterogeneous in their firing, as neighboring neurons often showed very different firing patterns. These findings are the counter to expected results and of those described above.
- A similar study by the same group has been performed on iPSC-derived neurons (114). They classify AP firing into the same four categories and find similar heterogeneity. Although no developmental analysis was done, this study compared a single control line and a single schizophrenia patient line. There were no significant differences between these lines in RMP, inward and outward currents, or input resistance. Input resistance was also much greater than previous studies by other groups (>2.5 GΩ) and RMP was much more depolarized (between −20 and −30 mV). Importantly for the field, this paper looked at spontaneous electrical activity including AP firing and the presence of UP-states, and later was able to monitor this activity with calcium imaging and correlate single spikes measured with patch clamping with single calcium transient *via* imaging on the same neuron. As single-cell PCR was performed on each cell, they were also able to screen for genes that correlate with the presence of UP-states.

The above studies have begun to establish a foundation of electrophysiological benchmarks for iPSC-derived neurons and have in some cases uncovered cellular phenotypes related to disease. However, these studies have not been able to measure these features in the context of a more physiological neural environment and generally focus on electrophysiological recordings from individual neurons without addressing synaptic input and network connectivity. Below we address some of the progress relate to these experimental issues.

## The Expanding Toolbox for iPSC-Derived Neurons

Functional characterization of neurons *via* electrophysiological analysis is important in light of the fact that neurons communicate *via* the release of neurotransmitter and AP firing. However, this functional input and output occurs in the context of brain microcircuits composed of distinct and defined cell types which are part of larger brain networks. Disease-induced dysfunction is often due to disruptions of particular cell types within brain circuits and networks. For this reason, it is important to attempt to study iPSC-derived neurons in similar contexts.

To these ends, some early progress has been made. This topic has been reviewed in-depth elsewhere (115), but cell type- and region-specific differentiation protocols are gaining more attention. Such protocols have been used to generate motor neurons (116), dopaminergic neurons (117), GABAergic neurons including specific subtypes (118), serotonergic neurons (119), hippocampal dentate granule neurons (120), and hypothalamic neurons (121). Protocols have also been established for generating astrocytes (122) and microglia (123), glial cell types that play important roles in synaptic development and pruning.

In addition to examining gene and protein expression of cell type-specific markers, several of these studies have undertaken detailed electrophysiological characterization. For example, one study carried out robust electrophysiological characterization of GABAergic neurons including AP firing, synaptic responses, synaptic integration in transplantation studies, and paired this with disease modeling (124). In other studies, iPSC-derived dentate gyrus granule neurons were generated from bipolar disorder (BD), as these cells have been previously tied to BD pathophysiology (125). This paper used a combination of gene expression studies, electrophysiology, and calcium imaging to establish a hyperexcitable phenotype in these neurons. Perhaps the more impressive finding in these studies is that neurons derived from patients who clinically responded to lithium treatment, also showed reduced hyperexcitability with lithium treatment, while non-responding patient neurons also had no response. This was recapitulated in the follow-up study in which DG neurons were generated from a different source of somatic cells from a separate cohort of BD patients (126).

As with animal models, manipulating specific cell types within relevant circuits with optogenetics is a crucial tool for advancing our knowledge of how elements of these circuits work. To this end, various studies have been published recently on optogenetic manipulation of hESC and iPSC-derived neuron activity. For example, a model circuit of motor neuron-to-muscle synapse was used to study Myasthenia Gravis (127), and it was shown that optogenetic stimulation of the motor neuron could result in muscle twitch. Similarly, human stem cell-derived neurons have been transplanted into animal brains where optogenetic stimulation of dopamine neurons was used to study Parkinson’s disease (128).

A similar transplantation study of iPSC-derived neurons paired with optogenetic stimulation has been used to study synaptogenesis and synaptic integration (129). These experiments showed that transplanted iPSC-derived neurons have more immature electrophysiological features compared with host cells, including slower APs and a more depolarized RMP. In addition, host cells formed limited functional synaptic connections with grafted cells in early development (as measured by optogenetic stimulation of host cells), but this integration increased by 24 weeks post-graft. It has also been shown that AP firing of iPSC-derived neurons can be controlled by optogenetic stimulation (107).

Several recent studies have explored approaches to promote neuronal and synaptic maturation. For example, the culture media most widely used for the maintenance of neuronal cell cultures have been generated to promote cell survival and neuronal differentiation, and it is not clear how well these media support neuron function and maturation of electrophysiological properties. One of the most comprehensive electrophysiological characterizations of iPSC-derived neurons to date found that traditional DMEM/F12 and Neurobasal media actually impair electrophysiological function and maturation, as measured by AP firing, excitatory and inhibitory synaptic activity, and voltage-gated currents (130). A novel medium was designed that promoted neuron function by eliminating ingredients from traditional media that were found to impair function. Both calcium imaging and MEA recordings were also used in this study to measure network activity of neuronal cultures.

It is clear that many distinct differentiation protocols can generate functionally mature neurons. However, the heterogeneity of neuronal electrical properties remains an issue in the field. To address this, a recent study classified neurons into subtypes *via* an unbiased statistical analysis of electrophysiological measurements including resting membrane potential, Na^+^ and K^+^ current properties, and AP properties (131). This approach allows the objective study of more homogenous and uniformly mature neurons. Importantly, this study found that the frequency of both spontaneous excitatory and inhibitory synaptic currents was much higher in more mature cells, and that soma size and dendritic complexity were also linearly related to neuronal maturity. Single-cell sequencing revealed that mRNA expression, including transcript levels associated with AP firing, synaptic function, channels, and synaptic receptors, all correlated with electrophysiological maturity. In addition, biomarkers were identified, including GDAP1L1, which predicted functional maturity based on electrophysiological properties.

Despite these early advances, many studies using iPSC-derived neurons lack thorough electrophysiological characterization. The study of actual function in these cells, especially when testing treatments to rescue dysfunction, is of the utmost importance. Thus, electrophysiological analysis must be a main focus of modeling neurological disorders with iPSCs in the future. This next generation of studies should also continue to pair electrophysiological analysis with newer protocols for cell type-specific cultures, as discussed, with combinations of neuronal types and more defined neural circuits. Pairing single-cell electrophysiology at this level with population analysis using calcium imaging or MEAs will also add another layer of understanding to many of these studies.

## The Way Forward

It is clear that synaptic impairments are a common feature of neurodevelopmental disorders. This pathophysiological hallmark has been observed in numerous mouse models and has been extensively studied using electrophysiology, as understanding the functional outcomes of these changes, especially within physiological neural circuits in the mouse brain, will better inform our understanding of disease. In line with this, we have discussed advances in iPSC-derived neuron technology and how electrophysiology and cell type-specific differentiation protocols are bringing us closer to similar levels of rigor of disease modeling in this new field. However, little work has differentiated whether any of these findings are truly primary disease phenotypes or either downstream or compensatory changes in response to disease. Outside of mouse models, studies of human postmortem brain tissue from ASD patients have reported changes in neuron size and number in various regions of neocortex, including prefrontal cortex, a cortical area important for higher-order cognitive processes including social, emotional, and communicative functions (132, 133). Such changes may be the result of deficient activity-dependent development of neuronal networks, a process heavily dependent on proper synaptic signaling in early nervous system development. In other words, processes commonly disrupted in mouse models of neurodevelopmental disorders. Thus, an appealing hypothesis for the pathophysiology of autism is one in which deficient spontaneous synaptic and AP firing activity, early in neural development, results in sparser and aberrant connectivity and changes to neuron morphology and neural circuitry. Abnormal connectivity in combination with impaired synaptic plasticity would result in neuronal networks that may insufficiently process synaptic input during activity-dependent synaptic remodeling, a window of development in which sensory input is critical in further developing and refining synaptic connections and neural networks (134). Interestingly, this marks the period of development when autistic features first emerge.

Although it is clear that autism and autism-associated neurodevelopmental disorders can be viewed as disorders of synaptic structure and/or function, this view does not account for heterogeneity between these clinically distinct syndromes, nor does it explain the heterogeneity from patient-to-patient within a single syndrome. In many cases, it is also unclear how synaptic deficits might lead to treatments for each of the specific autism-associated syndromes. This goal requires a better understanding of the primary pathophysiology of these disorders throughout the earlier stages of development before secondary synaptic deficits accumulate.

## A New Perspective for Understanding Synaptic Deficits

The studies reviewed above indicate widespread synaptic dysfunction among various clinically distinct neurodevelopmental disorders. However, it is not clear whether these synaptic deficits are primary cellular phenotypes directly downstream of genetic/molecular insults, secondary phenotypes, or the result of compensatory mechanisms in response to primary changes in excitability or network activity. This latter scenario is an interesting hypothesis in light of the limited progress that has been made in translating findings in mouse models to human ASD patients (11). As iPSC-derived neurons have been shown to be, at least transcriptionally, equivalent to mid- to late-gestational age, these cells provides a unique opportunity for investigating these important questions.

As described earlier, iPSC-derived neurons have been used to study SHANK3 mutations, which are associated with PMS. The finding that PMS neurons had a significantly elevated input resistance led to a follow-up study to investigate the possible causes for this. Analysis of hESC-derived neurons with either heterozygous or homozygous SHANK3 mutations at ~3-week *in vitro* replicated the increased input resistance reported in patient-derived PMS neurons, but also had decreased dendritic complexity, reduced synaptic event amplitudes, and increased AP firing (86). These findings were confirmed in SHANK3 mutant neurons from a mouse model. This change in synaptic activity and AP firing is reminiscent of many functional findings associated with studies of neurological disease, and, on its own, suggests no particular mechanism for separating PMS from other neurological disorders. The increased input resistance suggested decreased channel expression, and further experiments found that the hyperpolarization-activated current (Ih), which plays an important role in regulating neuronal excitability, was greatly diminished in SHANK3 mutant neurons. Moreover, SHANK3 interacts with the HCN channels that mediate Ih, suggesting that SHANK3 may act as a scaffolding protein for these channels. Most importantly, prolonged HCN channel block in control neurons recapitulated the entire spectrum of SHANK3 mutant phenotypes, suggesting that changes in excitability, synaptic activity, and dendritic morphology are either downstream or compensatory effects of HCN channel/Ih reduction (86).

We have recently published a study on the use of iPSC-derived neurons to study functional impairments of AS-derived neurons (52). Electrophysiological analysis over 20 weeks in culture revealed maturation of physiological properties including RMP hyperpolarization, increases in AP maturity, decreases in AP duration, increases in both AP amplitude and transient K^+^ current density, and increases in the frequency of synaptic activity as well as the percent of synaptically active cells. Spontaneous AP activity as assayed by calcium imaging also was present throughout *in vitro* development in control neurons. These features are more mature than many of those described in the studies above, although these neurons have been cultured for much longer, and are closest to those of iPSC-derived neurons that have been cultured in the presence of astrocytes. Thus, these data provide a robust timeline for electrophysiological maturation of iPSC-derived neurons that could inform future studies.

Compared with control neurons, AS-derived neurons failed to show the same degree of maturation across 20 weeks in culture. AS neurons showed immature resting membrane potentials and firing, and lower levels of synaptic activity. They also had significantly fewer AP-dependent calcium transients. Impaired maturation was seen in neurons derived from large deletion AS patients as well a patient with a mutation to *UBE3A* alone. To further confirm the critical role of *UBE3A* loss, a CRISPR-engineered *UBE3A* knockout line recapitulated the entire range of AS developmental phenotypes (52).

It is known from mouse models of AS that there is a critical period (early in development) in which rescue of *UBE3A* can also rescue behavioral phenotypes (135). To determine whether acute *UBE3A* changes cause similar phenotypes, we knocked down (KD) *UBE3A* using treatment with antisense oligonucleotides against *UBE3A* at early (6–9 weeks in culture) and late (18–20 weeks in culture), which reduced *UBE3A* message and protein ~50%. While both early and late *UBE3A* KD in control neurons could recapitulate the depolarization of RMP, only early knockdown could change AP firing and synaptic activity. This prompted us to ask whether or not *UBE3A* loss results in a primary change to RMP that then drives changes in firing and synaptic activity, as changes in resting potential can disrupt neuronal excitability and function and thus trigger compensatory changes. To test this, we depolarized control neurons with KCl for the duration of their development followed by electrophysiological analysis. As with AS and *UBE3A* KD/KO neurons, KCl-treated control neurons displayed the entire spectrum of AS-phenotypes, despite the presence of *UBE3A* (52).

These phenotypes are not observed in mouse models of AS, although there has been an observation of a hyperpolarized RMP later in development (136). Such differences might be due to the differences in the developmental time periods being studied in mouse models vs iPSC-derived neuronal cultures. Changes in synaptic activity and plasticity commonly observed at mature ages in mouse models of AS may be a consequence of earlier changes in RMP, as suggested in our study. Relevant targets of UBE3A that may relate to changes in synaptic activity and plasticity have been hard to identify. It is known that ubiquitin ligases are expressed in different cell types and change their targets in a brain region- and developmental stage-specific manner (137). Thus, relevant targets of UBE3A may need to be identified during critical developmental periods.

We have also recently characterized genotypic differences between iPSC-derived neurons from Dup15q patients vs. controls subjects (57). In contrast to AS-derived neurons, Dup15q neurons do not show disrupted RMP development, however, AP firing maturation is slightly delayed. These neurons, however, have increased synaptic event frequency and amplitude and disrupted synaptic plasticity, including the inability to scale down AMPAR amplitudes when challenged with increases in network activity by blocking GABARs. This latter plasticity is known as homeostatic synaptic plasticity and is important for maintaining a set point of neuronal firing and its disruption has been linked to neurodevelopmental disorders (138). In line with this, Dup15q neurons have increased spontaneous AP firing and increased synchrony compared with control neurons, as seen by patch clamp recordings and population calcium imaging. Finally, pharmacological screening, flow cytometry, and immunocytochemistry reveal that this excessive firing is due, at least in part, to disruption of seizure-associated KCNQ2 channels.

These results establish three mechanisms of hyperexcitabilty in Dup15q neurons; increased synaptic event frequency and amplitude, impaired synaptic scaling, and increased AP firing due to KCNQ2 disruptions. Although we have not yet differentiated primary vs. secondary phenotypes, it is interesting to speculate that the impaired down-scaling in Dup15q neurons may contribute to the development of excessive firing and synchronous activity. Likewise, impaired up-scaling may be occluded by excessive baseline firing. Deficits in KCNQ2 channels may also contribute to synaptic scaling deficits.

As discussed earlier in this review, iPSC-derived neurons from patients with Dravet syndrome express increased sodium current density and increased AP bursting in both pyramidal (excitatory) and bipolar (inhibitory) neurons (103). This phenotype also includes bursting activity in both types of neurons from these patients. Although hyperexcitability in pyramidal neurons would contribute to seizure susceptibility, the same phenotype in presumably inhibitory neurons would inhibit seizure activity. It has also been shown both in human (104) and mouse (139) that sodium currents are actually unchanged in excitatory neurons, and decreased in inhibitory neurons. This net disinhibition is more consistent with seizure activity. Despite these contrasting results, the initial report of paradoxical sodium current density changes were later confirmed in a mouse model at a developmental age that had not been previously investigated (140). Although the combination of these studies does not directly address primary vs. secondary phenotypes, it does suggest the increases in sodium current density observed by Liu et al. and Mistry et al. may actually be the earliest signs of dysfunction that may eventually lead to the contrasting findings reported by other studies.

## Limitations and Future Directions

Despite their great promise and the important advancements made using iPSC-derived neuron models, the limitations and road blocks that lie ahead are many. These shortcomings have been extensively reviewed elsewhere (141), but there are two specific areas worth discussing further, in light of identifying primary physiological phenotypes in these models: (1) synaptic plasticity and (2) neural circuitry.

Plasticity is the process by which neurons undergo changes in either structure, function, or both, in response to various stimuli including changes in synaptic input and network activity or cell-autonomous changes like intrinsic excitability. In line with this broad definition, a large number of functionally distinct types of plasticity have been established and have been reviewed at length (142). Briefly, one broad type of plasticity, activity-dependent plasticity, includes processes that both strengthen and weaken synaptic strength, termed LTP and long-term depression (LTD), respectively. This type of plasticity is important for the proper development and refinement of cortical circuits and is critical to normal brain function. In line with this, it has been well established that disruptions of these types of plasticity are important hallmarks of a broad range of neurological disorders including Alzheimer’s disease and schizophrenia, as well as autism-associated neurodevelopmental disorders such as Fragile X, Rett syndrome, Tuberous sclerosis, and AS (15). Although the mechanisms underlying these types of plasticity have been extensively studied in mice, similar types of synaptic plasticity have not been established in iPS-derived neuron models to date. This remains a critical area for further study (143). Despite the lack of reports of synaptic plasticity in stem cell-derived neurons, there have been promising steps forward in a limited number of studies. For example, iPSC-derived neurons cultured on MEAs showed LTP and LTD-like phenomena as measured by changes in spiking activity in response to high frequency stimulation (144). Although these changes occurred in different neurons in response to the same stimulation, these phenomena lasted for at least 1-h post-induction. How this type of plasticity relates to changes in synaptic function or structure was not investigated.

Another common form of plasticity is induced by long-term changes in network activity. Blocking AP firing (with TTX) or increasing AP firing (*via* GABA block) results in changes to AMPA receptor expression. Such plasticity, termed homeostatic synaptic scaling, has also been demonstrated in neurons derived from a human embryonic stem cell line (145). Deficits in synaptic scaling have been tied to various neurodevelopmental syndromes in mouse models. In a study of Dup15q using human iPSC-derived neurons, control cells displayed the expected increases and decreases in AMPA event amplitude in response to manipulations in network activity as measured by both immunocytochemistry and electrophysiology. Interestingly, Dup15q neurons were not able to scale AMPA events either up or down, which could contribute to a hyperexcitable phenotype in these cells (57).

Finally, pharmacologically induced plasticity protocols, which have successfully been used in mouse brain slices and cultured neurons to elicit LTP (146), have also been used in studies of iPSC-derived neurons (52). In a study using AS neurons, for example, plasticity induction *via* pharmacologically increased cAMP levels and enhanced activation of NMDA receptors caused a long-term elevation of spontaneous synaptic event frequency in control neurons which was absent in AS-derived neurons, paralleling LTP deficits in AS mouse models. Similar plasticity deficits were seen in the Dup15q neurons, and in both cases, the deficits in plasticity of synaptic transmission were paralleled by deficits in plasticity of AP firing as revealed by calcium imaging.

As mentioned, synaptic plasticity plays an important role in the function of neuronal circuits in the brain, and many experimental protocols rely on the established circuits of the brain for recording from neurons in a particular area or layer of the brain and stimulating neurons in another area or layer. Another important limitation of typical iPSC-derived neuronal cultures is the lack of a layered cortical structure. Development of 3D culture systems will be important for studying human neurological disorders and for understanding how disease-relevant cellular phenotypes are expressed in an anatomically accurate cortical or hippocampal circuit.

Despite this limitation, progress has been made in recent years in establishing protocols that yield 3D brain-like structures called “organoids” that have generated a proof-of-principle for the *in vitro* study of important neurodevelopmental processes in models of, for example, ASD (91), lissencephaly (147), microcephaly (148), and ZIKA virus infection (149–151). These advancements have been extensively reviewed elsewhere (152–155). It is worth mentioning, however, that many of these studies, although using different protocols, have shown expression of brain region-specific markers, appropriate layering, interneuron migration, the presence of outer radial glia (an important human cell type), migration of cortical neurons, radial organization, presence of active zones, and the presence of many of the correct cell types, including astrocytes. Moreover, many of these papers also pair this characterization with electrophysiological analysis and gene expression analysis, providing a rich data set for identifying underlying disease pathophysiology.

## Conclusion

Since the discovery of somatic cell reprogramming and the differentiation of patient-specific neural tissue almost a decade ago, we have come a long way in developing this powerful technology, and the possibilities to use this platform for understanding human disease and developing targeted therapies remains wide open. Despite this, challenges remain and overcoming these obstacles will certainly be a prime focus of investigation moving forward. Although animal models have been important for gaining insight into disease mechanisms and relating disease phenotypes to animal behavior, there has been little translational success of these findings to treatments for human patients. One important issue relates to studying the earliest stages of neural development, to better establish and understand the initiation of pathophysiology without the confounding influences of homeostatic responses and the development of secondary phenotypes. In relation to human tissue, there is great difficulty in assessing primary phenotypes from postmortem brain samples, unhealthy tissue, or tissue collected later in development (156). These specific cases are confounded by compensatory changes of neurons and other cell types to the presence of abnormal tissue or disrupted cell types. Also, these cases miss the period during which synapses are pruned and excessive neurons undergo apoptosis to refine neural circuits.

Another concern, which has also been commented on elsewhere, focuses on the differences in the development of the brain between mice and humans. Such matching of developmental stages is difficult, and studying mouse models at ages which reflect distinct stages of human gestation and brain development is challenging (157). The combination of these situations lends further support to advantages of human stem cell-derived neurons. Regardless of their current limitations, iPSC-derived models provide a unique opportunity to address these important questions. These studies will drive new knowledge as it relates to disease pathophysiology which can be used to further inform experiments using animal models. This will provide a powerful cross-talk between model systems which promises to lead to a much more robust understanding of disease mechanism that can be leveraged to better develop molecular targets for treatment of human disease.

## Author Contributions

The first draft was written by JF and subsequently edited and approved for publication by JF and EL.

## Conflict of Interest Statement

The authors declare that the research was conducted in the absence of any commercial or financial relationships that could be construed as a potential conflict of interest.
